# Polymethylmethacrylate Pulmonary Embolism Following Kyphoplasty

**DOI:** 10.5811/cpcem.2019.4.42324

**Published:** 2019-05-20

**Authors:** Oliver Morris, Josephin Mathai, Karl Weller

**Affiliations:** St. Lucie Medical Center, Department of Emergency Medicine, Port St. Lucie, Florida

## Abstract

We report a case of polymethylmethacrylate cement pulmonary embolism (PE) that occurred two days following a minimally invasive kyphoplasty procedure. Our patient developed non-specific rib pain postoperatively followed by dyspnea, prompting presentation to the emergency department. The polymethylmetacrylate cement was visualized on initial chest radiograph and further characterized using computed tomography. The patient was admitted and anticoagulation started, later having an uncomplicated hospital course. The polymethylmethacrylate cement has a well-documented history of leakage and other postoperative complications. Cement PE, while rare, can present similarly to a thrombotic PE and requires adequate long-term anticoagulation with close follow-up.

## INTRODUCTION

Vertebral compression fractures make up approximately one half of all osteoporotic fractures in the United States (U.S.), affecting over 700,000 people per year.^1^ Patients with compression fractures often experience severe pain that may limit mobility, increase morbidity, and can be a significant source of healthcare resource utilization. Multiple treatment modalities have been used including medical management, pain management, physical therapy, bracing, and surgery. The surgical therapies consist of minimally invasive techniques such as percutaneous balloon kyphoplasty and vertebroplasty, where a cement polymer is injected into the vertebrae to stabilize the osseous structure. There are estimated to be over 25,000 kyphoplasty and vertebroplasty procedures performed in the U.S. each year, and they can be associated with severe intra- and postoperative complications.^2^ This case report highlights one of the rarer but often more severe complications, polymethylmethacrylate (PMMA) pulmonary embolism (PE).

## CASE REPORT

A 43-year-old male construction worker with a history of chronic back pain and recent kyphoplasty two days prior, presented to the emergency department (ED) for the second time that day for dyspnea. The patient had been seen in the ED earlier in the day by another provider for nonspecific lower back and flank pain that was medically treated with improvement of symptoms. A few hours after arriving home, the patient became dyspneic and returned to the ED for evaluation.

On physical examination, he appeared to be mildly tachypneic. His blood pressure was 105/71 millimeters of mercury, pulse 86 beats per minute (BPM), respiratory rate 20 breaths per minute, and oxygen saturation of 95% on room air. He did not appear to be in respiratory distress with no accessory muscle use. Lungs were clear to auscultation but mildly diminished. He exhibited no wheezing, rhonchi, or rales. The heart sounds were regular, with no audible murmur. Abdomen was soft and nontender, with positive bowel sounds. There was no midline spinal tenderness. He had several well-healing, non-erythematous paraspinal puncture wounds from the kyphoplasty procedure two days prior. The rest of his physical exam was unremarkable.

**Image 1:**
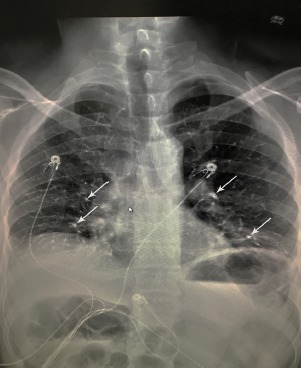
Chest radiograph of a 43-year-old male depicting multiple hyperdense opacities (arrows) with vascular crowding and atelectasis at lung bases.

**Image 2:**
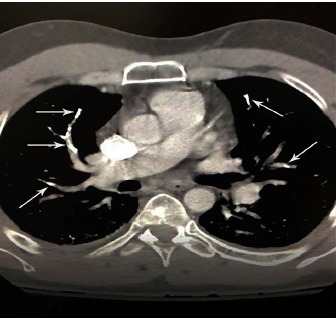
Computed tomographic angiogram of the chest of a 43-year-old male depicting hyperdense material in distal pulmonary arteries (arrows) consistent with cement emboli.

Initial workup consisted of basic metabolic panel, complete blood count, troponin, electrocardiogram (ECG) and a chest radiograph (CXR). When we applied the Wells criteria for PE, the patient scored 1.5 for having had a surgery in the previous four weeks. This score put him in the low-risk group with a 1.3% chance of PE.^1^ The ECG showed a normal sinus rhythm at 85 BPM. The CXR revealed pulmonary cement embolism with mild vascular crowding and atelectasis at the lung bases (Image 1). With this finding, a computed tomography angiography of the chest was ordered, which revealed cement in distal pulmonary arteries consistent with cement emboli along with patchy, ground-glass opacity worrisome for infiltrate (Image 2). The patient was immediately treated with heparin and admitted to the hospital for continued management. While there, he was treated according to guidelines for thrombotic PEs and started on six-month warfarin therapy. He was discharged home two days later.

**CPC-EM Capsule**

What do we already know about this clinical entity?

Polymethymethacrylate pulmonary embolism is a rare but known complication of kyphoplasty. It has been reported in orthopaedic literature but rarely in emergency medicine.

What makes this presentation of disease reportable?

There have been very few reports of this disease entity in emergency medicine literature, so increasing awareness of post-kyphoplasty complications is essential.

What is the major learning point?

The novelty of this disease, along with the morbidity and mortality if left untreated, makes early recognition important.

How might this improve emergency medicine practice?

Early recognition of polymethylmethacrylate pulmonary embolism can lead to better patient outcomes.

## DISCUSSION

Kyphoplasty and vertebroplasty are two common surgical techniques used in stabilization and repair of vertebral compression fractures. The procedures are similar in that they use a cement, such as PMMA, which is injected into the vertebral body and allowed to harden. Kyphoplasty differs by first employing a balloon that is inflated in the vertebral body prior to the cement injection. This allows for height restoration of the affected vertebrae. The procedures themselves are minimally invasive, but the efficacy of kyphoplasty and vertebroplasty in osteoporotic vertebral fractures continues to be controversial. Two randomized, placebo-controlled trials found no significant benefit over conservative management.^2^,^3^,^4^,^5^ In 2010, as part of its clinical practice guidelines, the American Academy of Orthopaedic Surgeons strongly recommended against vertebroplasty for patients who present with an osteoporotic spinal compression. Since taking that stance, there have been several newer, unblinded trials and meta-analyses published that contradict the initial findings._._^6^,^7^,^8^

Cement extravasation is the most common and well-known complication of both vertebroplasty and kyphoplasty with rates as high as 41% and 18%, respectively.^9^,^10^ This leakage can cause damage to surrounding nerve and tissues, irritation of nerve roots, PE, and even reports of cardiac tamponade. The literature research revealed that the risk of PE ranges from 3.5–23%, with vertebroplasty leakages being more common and more significant._._^11^ Treatment in these cases has not been well defined, but the consensus is to proceed according to guidelines of thrombotic PEs. Initial heparinization and six months of continuous warfarin therapy is recommended in symptomatic peripheral and asymptomatic central PE along with admission for clinical observation and close follow-up. In rare instances of central symptomatic PE, surgical embolectomy may be considered._._^11^

## CONCLUSION

This report highlights the importance of recognizing cement PE in a postoperative kyphoplasty patient presenting for non-specific chest complaints in an otherwise healthy individual and minimal PE risk factors.

## References

[1] McCall T, Cole C, Dailey A (2008). Vertebroplasty and kyphoplasty: a comparative review of efficacy and adverse events. Curr Rev Musculoskelet Med.

[2] Laratta JL, Shillingford JN, Lombardi JM (2017). Utilization of vertebroplasty and kyphoplasty procedures throughout the United States over a recent decade: an analysis of the Nationwide Inpatient Sample. J Spine Surg.

[3] Buchbinder R, Golmohammadi K, Johnston RV (2015). Percutaneous vertebroplasty for osteoporotic vertebral compression fracture. Cochrane Database Syst Rev.

[4] Savage JW, Schroeder GD, Anderson PA (2014). Vertebroplasty and kyphoplasty for the treatment of osteoporotic vertebral compression fractures. J Am Acad Orthop Surg.

[5] Klazen CA, Lohle PN, Vries J (2010). Vertebroplasty versus conservative treatment in acute osteoporotic vertebral compression fractures (VERTOS II): an open-label randomised trial. The Lancet.

[6] Guarnieri G, Masala S, Muto M (2015). Update of vertebral cementoplasty in porotic patients. Interv Neuroradiol.

[7] Yuan WH, Hsu HC, Lai KL (2016). Vertebroplasty and balloon kyphoplasty versus conservative treatment for osteoporotic vertebral compression fractures: A meta-analysis. Medicine (Baltimore).

[8] Yaltirik K, Ashour AM, Reis CR (2016). Vertebral augmentation by kyphoplasty and vertebroplasty: 8 years experience outcomes and complications. J Craniovertebr Junction Spine.

[9] Papanastassiou ID, Filis A, Gerochristou MA (2014). Controversial issues in kyphoplasty and vertebroplasty in osteoporotic vertebral fractures. Biomed Res Int.

[10] Papanastassiou P, Phillips FM, Van Meirhaeghe J (2012). Comparing effects of kyphoplasty, vertebroplasty, and non-surgical management in a systematic review of randomized and non-randomized controlled studies. Eur Spine J.

[11] Krueger A, Bliemel C, Zettl R (2009). Management of pulmonary cement embolism after percutaneous vertebroplasty and kyphoplasty: a systematic review of the literature. Eur Spine J.

